# Hereditary Hemorrhagic Telangiectasia With Multiple Ear, Nose, and Throat (ENT) Manifestations: A Case Report

**DOI:** 10.7759/cureus.42706

**Published:** 2023-07-30

**Authors:** Eleni Litsou, Lentiona Basiari, Georgios Tsirves, Georgios V Psychogios

**Affiliations:** 1 Department of Otorhinolaryngology, Head and Neck Surgery, University Hospital of Ioannina, Ioannina, GRC

**Keywords:** arteriovenous malformations, recurrent epistaxis, telangiectasias, rendu-osler-weber syndrome, hereditary hemorrhagic telangiectasia

## Abstract

Hereditary hemorrhagic telangiectasia (HHT), also known as Rendu-Osler-Weber syndrome, is a rare autosomal dominant multisystem disorder. It is a mucocutaneous and fibrovascular dysplasia, the diagnosis of which is based on the fulfillment of the four Curaçao criteria: 1) recurrent epistaxis; 2) dermatovascular mucosal telangiectasias at characteristic sites: skin of the face, ears, fingertips, lips, tongue, and oral and nasal cavity; 3) arteriovenous malformations (AVMs) of visceral organs and central nervous system; and 4) family history: diagnosis of HHT in a first-degree relative. We describe a case of a 76-year-old patient who presented to our department with clinical manifestations of HHT in the skin (face, fingertips), lips, hard palate, tongue, ears, and nasal cavities. Individual and family history was obtained, as well as clinical laboratory examination, pan-endoscopy of the ear, nose, and throat (ENT) systems, and treatment of active foci of bleeding from the above areas. The otolaryngologist may be the first doctor to suspect Rendu-Osler-Weber syndrome and the one responsible for treating patients with HHT since recurrent epistaxis is the most frequent (90-96% of patients) and the earlier manifestation of the disease and the main reason for the arrival of these patients in the Emergency Department. The purpose of this study is to present a clinical case of Rendu-Osler-Weber syndrome with multiple ENT manifestations, as well as a review of the literature on their management and treatment.

## Introduction

Hereditary hemorrhagic telangiectasia (HHT) is a hereditary disorder of blood vasculature, which is characterized by the formation of multiple mucocutaneous telangiectasias and arteriovenous malformations (AVMs) within the pulmonary, cerebral, and gastrointestinal vasculature [1]. In the area of the lesions, there is an absence of intervening capillaries, resulting in the escape of venous blood due to a direct artery-to-vein connection [2]. Small lesions that are the size of a pinhead and pink to red in color are called telangiectasias, while larger lesions of a few millimeters to several centimeters are called AVMs.

The prevalence of Rendu-Osler-Weber syndrome varies between 1.5 and 2:5,000-10,000 persons worldwide, depending on the geographic area [3], with the highest incidence (one in 1331) in certain populations, including the Afro-Caribbean people of Curaçao and Bonaire regions. It is estimated that the overall prevalence of this disease is quite higher given that the signs of HHT may go unrecognized and untreated for decades or cause premature death [3].

The clinical diagnosis of Rendu-Osler-Weber syndrome is based on the presence of the Curaçao criteria [2]. It is considered "possible" when only two of the four criteria are present and "definite" when three of the following criteria are present:

1) Epistaxis: spontaneous and recurrent. It is due to telangiectasias of the nasal mucosa. It is the most frequent and earliest manifestation of the disease. They are usually nocturnal. The average age of the first appearance is 10 years, and its incidence increases with age. Specifically, 90-96% of patients with Rendu-Osler-Weber syndrome will eventually develop recurrent epistaxis [4]. In some cases, a hemodynamic disorder may coexist due to the acute loss of a large amount of blood. Patients report some special triggers, such as emotional state, dietary factors, and seasonal variations [5].

2) Telangiectasias: mucocutaneous, multiple, commonly seen on the skin (face, fingertips), lips, tongue, and oral and sinonasal cavity. They are found in 74% of patients, half of whom are under 30 years of age. Their number is increasing with age. The age of appearance of telangiectasias is 5-30 years later than epistaxis [4]. The morphology of telangiectasias varies from small, razed, stellate lesions to large, lifted agglomerated lesions.

3) Gastrointestinal (GI) tract, pulmonary, hepatic, cerebral, or spinal arteriovenous malformations.

The need for a multidisciplinary approach is underlined by the broad range of all organs that are affected in patients with HHT. Patients who have been diagnosed with pulmonary AVMs are at an increased risk of developing complications, such as brain abscess, stroke, and myocardial ischemia. These complications are caused by a paradoxical embolism, which can be septic, air-associated, or due to blood clots. To mitigate these risks, it is recommended that patients receive antibiotic prophylaxis prior to procedures that may cause bacteremia and avoid engaging in scuba diving activities. Additionally, healthcare providers should ensure that patients with AVMs undergo long-term follow-up to monitor the growth of untreated lesions or reperfusion of treated AVMs. In patients with HHT, hepatic AVMs are commonly present in 40-70% of cases. However, medical intervention is only necessary for patients who experience symptoms such as heart failure, pulmonary hypertension, abnormal cardiac biomarkers, abnormal liver function tests, abdominal pain, portal hypertension, or encephalopathy. These patients should be managed and closely monitored by specialized medical centers.

4) Positive family history: diagnosis of HHT in a first-degree relative.

Specifically, 90% of patients will show the above criteria by the age of 40 [4].

The clinical diagnosis can be confirmed by genetic testing of the patient. The pathogenesis of HHT has been linked to mutations in genes that are associated with the signaling pathway of the transforming growth factor-beta (TGF-b) superfamily. However, 98% of cases are caused by mutations of two genes on chromosome 9: endoglin (ENG) for type 1 (HHT-1) and activin Α receptor-like kinase (ACVRL1/ALK1) for type 2 (HHT-2), in 61% and 37% of cases, respectively [6]. Another gene, MADH4 (mothers against decapentaplegic homolog 4) on chromosome 18, is associated with a combination of both juvenile polyposis and HHT (JP/HHT), seen in approximately 1-2% of patients [1]. Lately, mutation in bone morphogenetic protein-9 (BMP9) has been related to a vascular anomaly syndrome phenotypically similar to HHT. The gene mutations encode proteins that modulate TGF-b involved in angiogenesis, which transmits signals to vascular endothelial cells, causing an inadequate response to angiogenic stimuli in specific locations. The remodeling of the vascular endothelium in mucosal vessels happens in an unregulated manner, resulting in the loss of flexibility and the expansion of arteriole-venule connections. Consequently, delicate and thinly walled telangiectasias develop within the nasal cavity in areas that are exposed to high airflow, which makes them susceptible to dryness or repeated mechanical injury. Recurrent spontaneous epistaxis results from traumatic damage to the vessel wall, which lacks elastic and contractile elements. Thus, the discontinuity of the vassal walls at the sites of dysplasia and not the blood coagulation disorders is what causes the bleeding in patients with Rendu-Osler-Weber syndrome [2]. There are distinct phenotypic variations in HHT1 and HHT2 [6]. Patients with HHT1 are more likely to develop epistaxis and pulmonary AVMs early in life. HHT2 patients are more prone to hepatic AVMs. Genetic testing may not be positive in all people with HHT due to unrecognized gene mutations. Positive genetic tests can provide a definitive diagnosis, although they are expensive and are only recommended in certain circumstances, such as prenatal screening or index cases, and for members of families with known HHT who have a few symptoms established, or can exclude a definitive diagnosis [1].

## Case presentation

**Figure 1:**
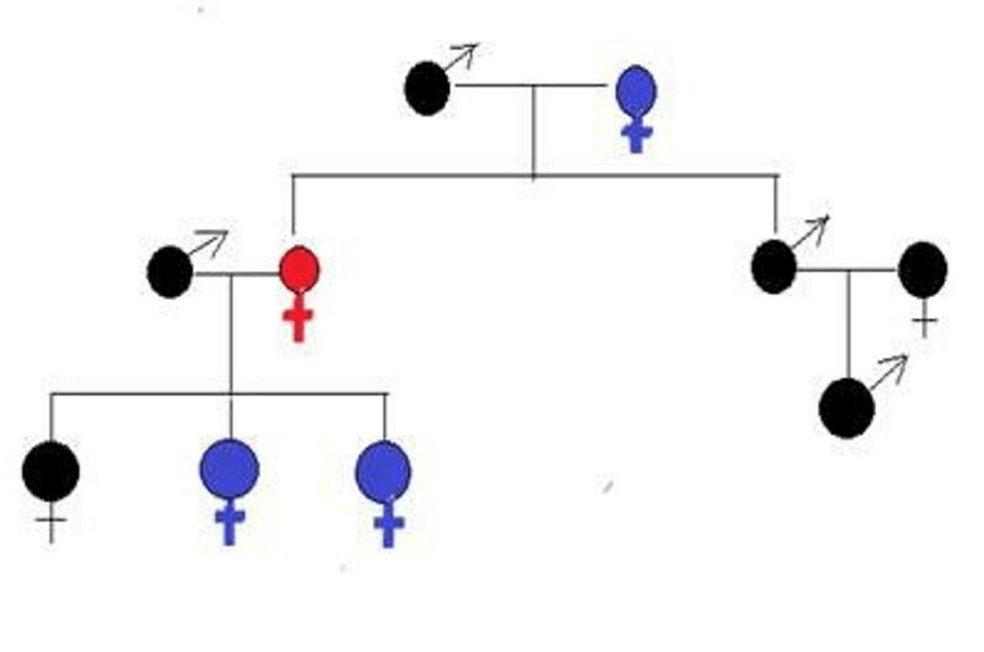
Family history of hereditary hemorrhagic telangiectasia (HHT) in first-degree relatives; reported case in red, affected members in blue, and non-affected members in black color

A 76-year-old patient came regularly (2-3 times every month) to our Emergency Department with spontaneous and uncontrolled bleeding from the hard palate and nose, associated with a significant microcytic iron deficiency anemia that required transfusions. The patient reported almost daily epistaxis, usually nocturnal, which has been controlled with topical means. HHT was diagnosed clinically since the patient met all four Curaçao criteria, namely:

(1) Family history of the disease in first-degree relatives (Figure 1).

(2) Presence of mucocutaneous telangiectasias on the inner surface of the hands, facial skin, lower lip, tongue, hard palate, nasal cavities, and on the tympanic membrane (Figures 2-9).

**Figure 2:**
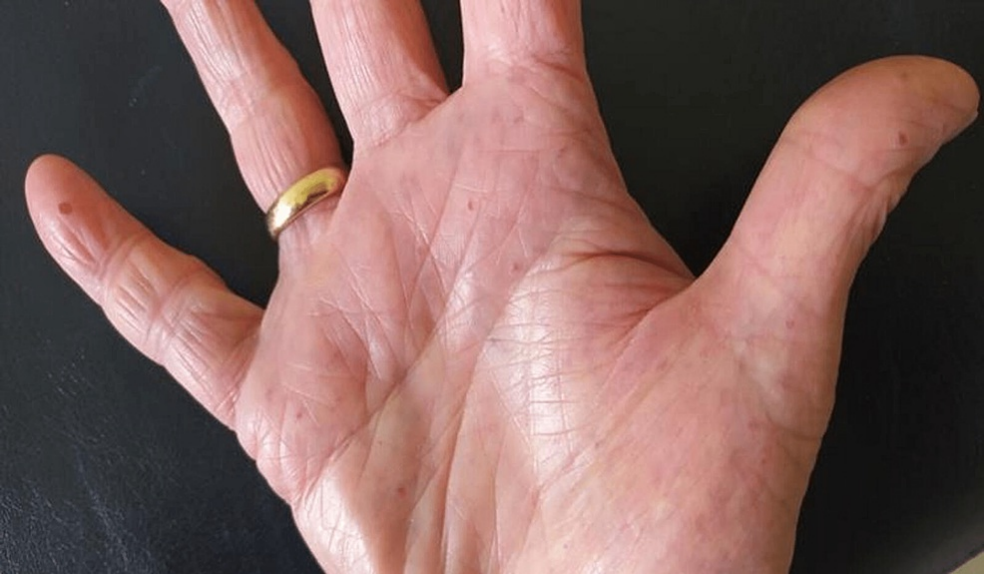
Multiple telangiectasias on the inner surface of the hand

**Figure 3:**
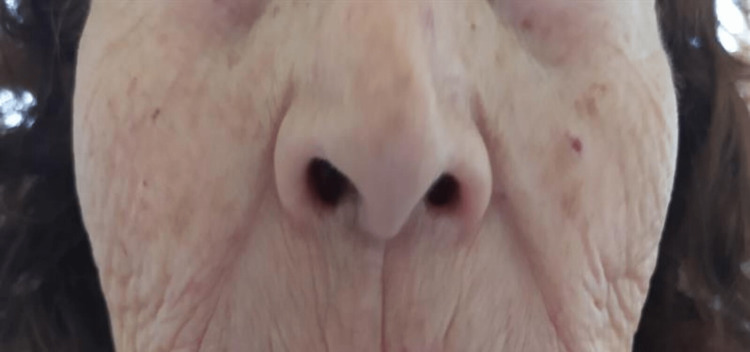
Facial skin telangiectasias

**Figure 4:**
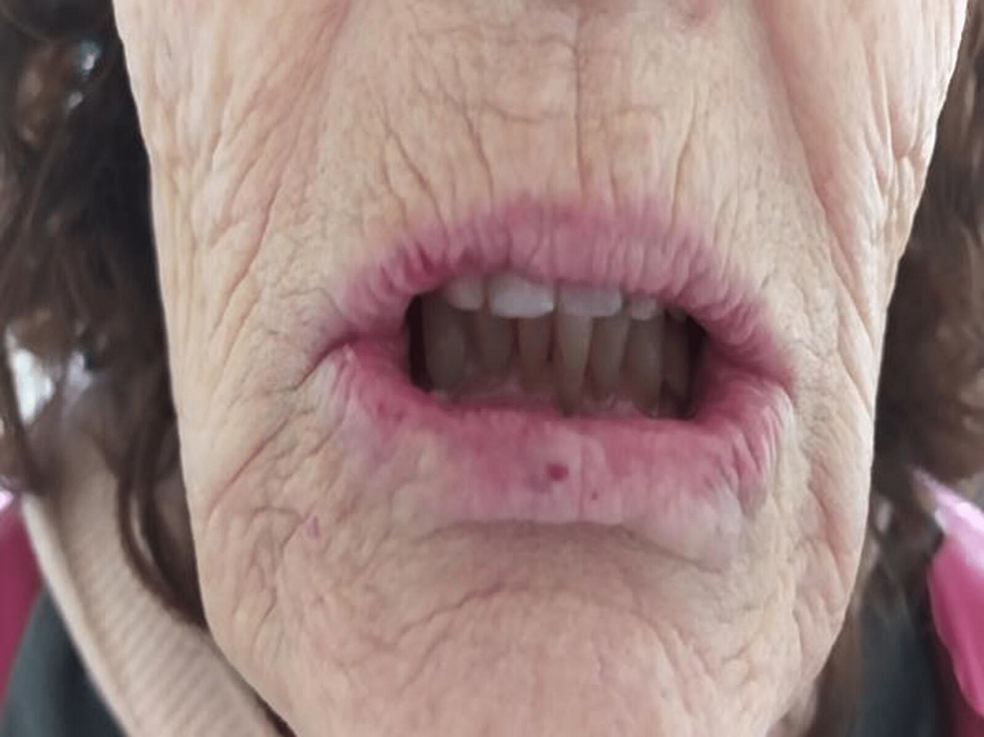
Lower lip telangiectasias

**Figure 5:**
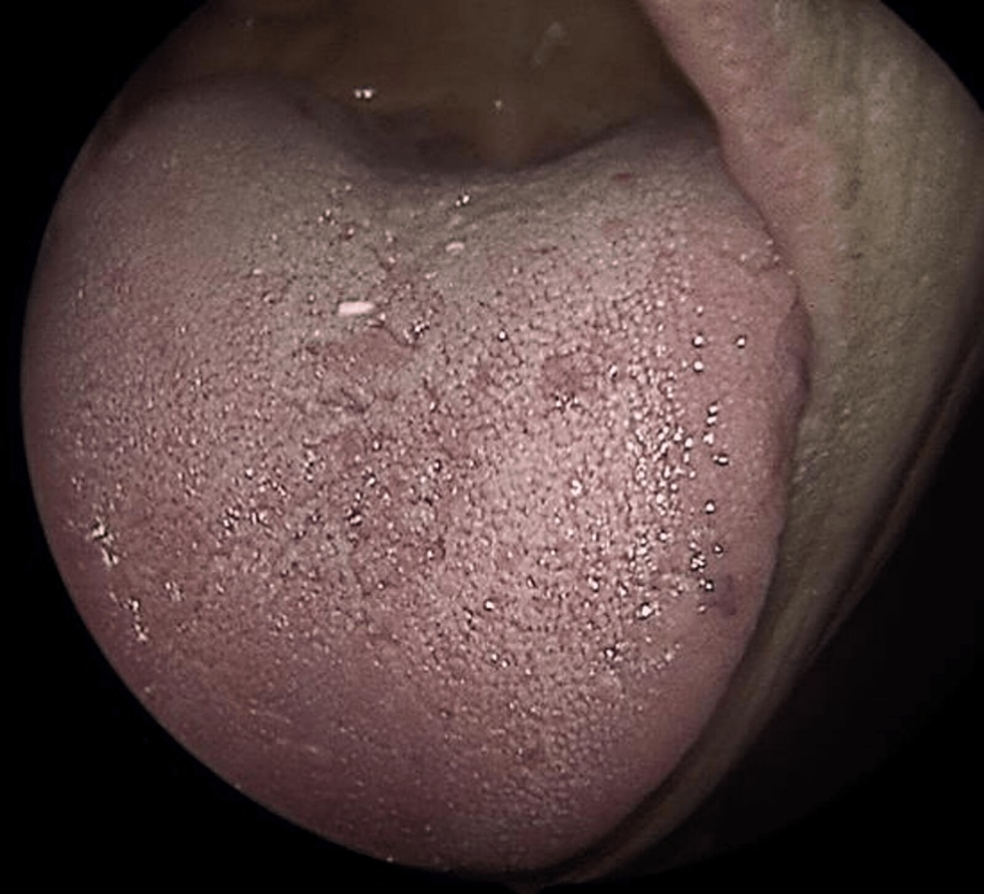
Tongue telangiectasias

**Figure 6:**
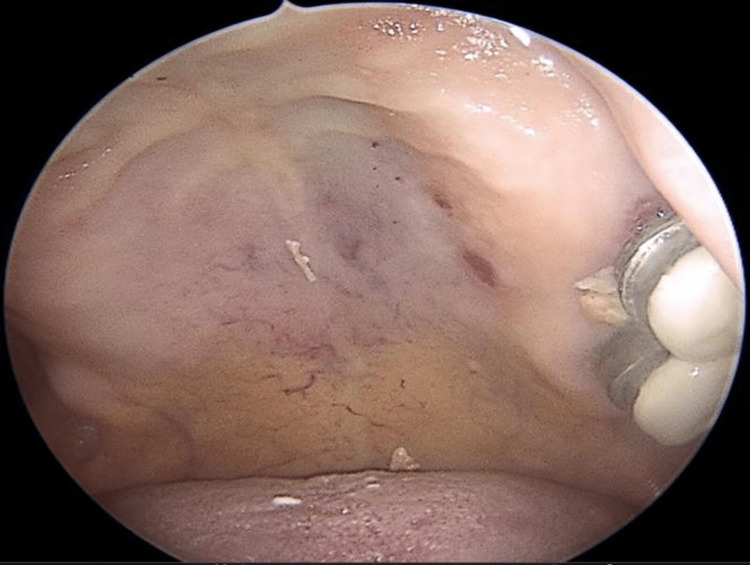
Telangiectasias on the oral mucosa (hard palate)

**Figure 7:**
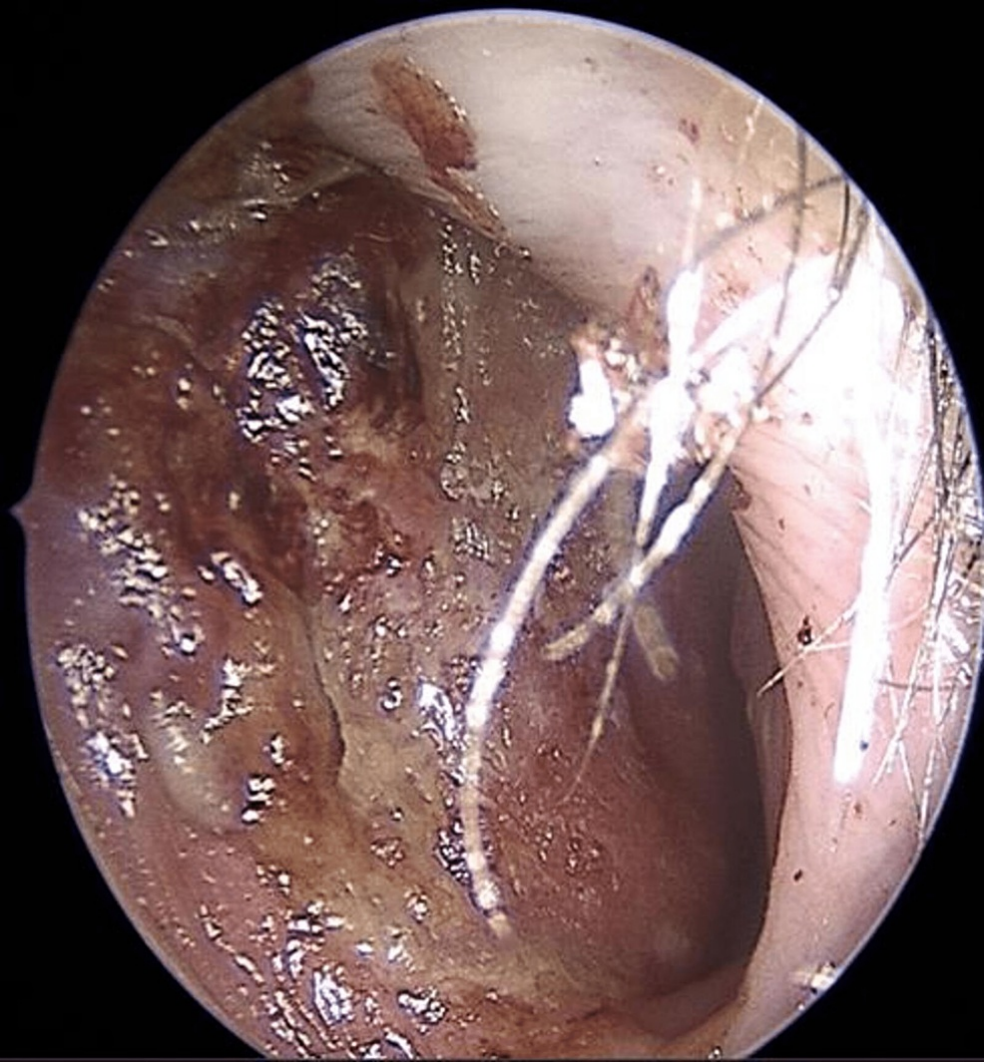
Endoscopic view of the left nasal cavity exhibiting Shapshay type II (large, diffuse, interconnecting) telangiectasias in the nasal septum

**Figure 8:**
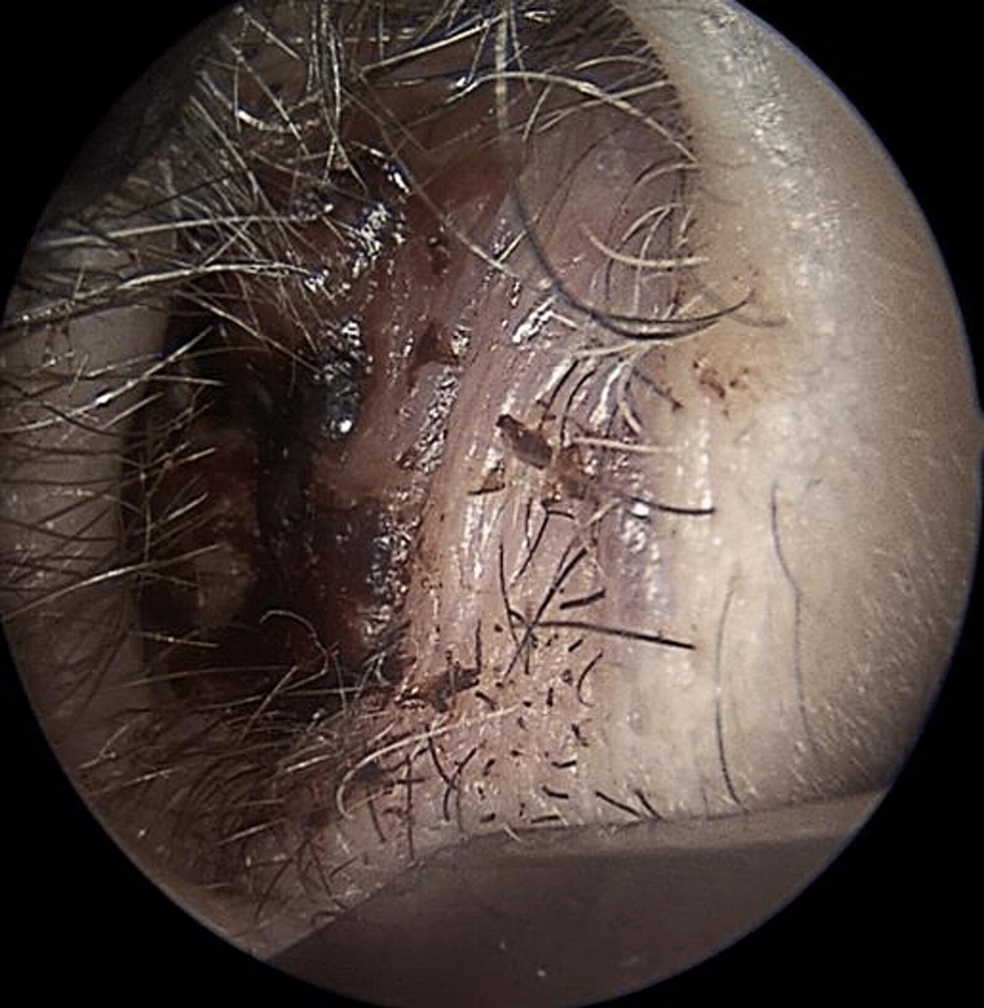
Endoscopic view of the right nasal cavity exhibiting telangiectasias and nasal crustings

**Figure 9:**
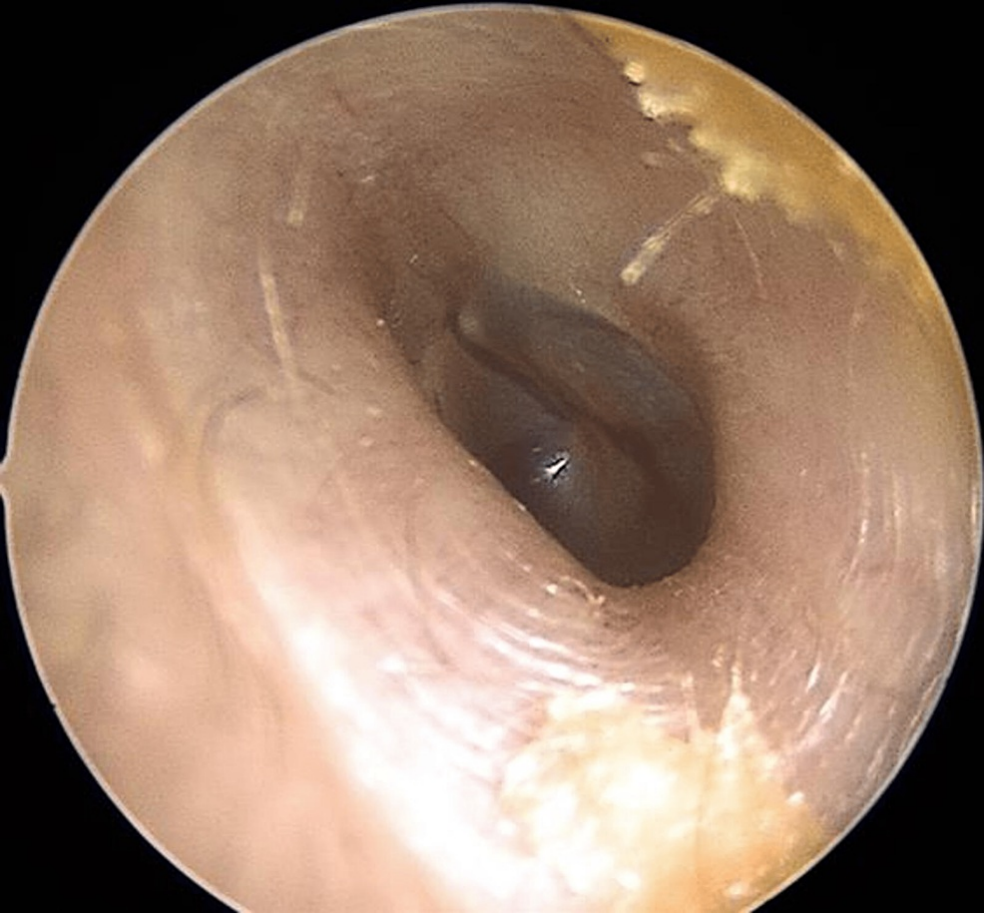
Right ear tympanic membrane telangiectasias

(3) Spontaneous and recurrent epistaxis.

(4) Pulmonary and cerebral AVMs on high-definition chest computed tomography and on brain magnetic tomography.

The rest of the patient's medical history includes the following: arterial hypertension, iron deficiency anemia, pulmonary hypertension, severe tricuspid and aortic valve insufficiency, moderate mitral valve stenosis, severe right ventricular and left atrium dilatation, placement of a permanent artificial pacesetter due to chronic arrhythmias, and dyslipidemia. The management of each episode of bleeding included immediate and rapid objective ear, nose, and throat (ENT) examination to identify the focus and to assess the severity and degree of bleeding. In any case, our main concern was a small and limited number of bloody procedures and manipulations, with the aim of reduced blood loss and prevention of new bleeding. Anterior rhinoscopy and nasal endoscopy revealed telangiectasia, and a careful and atraumatic examination was performed to prevent bleeding. A cotton ball soaked with oxymetazoline or an equivalent topical decongestant is a useful adjunct during the examination. Intravenous antibiotic prophylaxis, analgesics, sedatives, and antihypertensive treatment were administered before any scheduled procedure. Laboratory tests before surgery include a complete blood count, blood type, and crossmatch. The patient was asked for recent blood transfusions needed and was prepared for a possible transfusion during surgery. Further management included diligent preoperative, intraoperative, and postoperative local hemostasis with the instillation of Transamin (tranexamic acid that inhibits fibrinolysis and has an antihemorrhagic effect) in neurosurgical patties and placing them on the bleeding foci to be cauterized. This was followed by local anesthesia with cotton swabs sprayed with 10% xylocaine spray (lidocaine hydrochloride) for about 10 minutes and cauterization of the bleeding foci using bipolar diathermy. The order in which the bleeding sites were treated (hard palate, right and left nasal cavity) was based on the severity of the bleeding and the need for blood transfusion. The simultaneous treatment of both nasal cavities was not chosen to avoid the risk of perforating the nasal septum. In all bleeding foci that were treated, the postoperative course was uneventful without recurrence. The patient was given the following discharge instructions:

(1) Using a humidifier to maintain the air with sufficient humidity to prevent dryness of the nasal or oral mucosa and reduce the risk of bleeding.

(2) Maintaining nasal and oral hygiene. When it comes to preventing crusting and dryness, nasal saline irrigations have shown effectiveness. However, it is important to administer them with caution and in a gentle manner to avoid potential nosebleeds.

(3) Maintaining the oiliness of the nasal mucosa by using a special ointment.

(4) Avoiding triggers.

## Discussion

HHT is a progressively evolving disease, characterized by a wide range of symptoms, which show a correlation with age [4], affected organ, type, and extent of the attack.

The most prevalent, recurring, and initial indication of Rendu-Osler-Weber syndrome is the occurrence of spontaneous epistaxis caused by the presence of telangiectasias on the nasal mucosa. It first appears at a young age [3,4]. Almost all patients with HHT have been exposed to recurrent epistaxis sometime in their lives [1,4], some more frequently with several episodes per day and others more infrequently with occasional episodes over many years. In general, the incidence and severity of epistaxis increase with age [4]. In most patients, the aim of treatment is to reduce the frequency, volume, and severity of epistaxis and improve quality of life. Less frequently, the goal of treatment is to alleviate severe anemia or prevent life-threatening epistaxis. There is no standard treatment algorithm; however, a stepwise treatment strategy is recommended, starting with conservative local treatment and progressing to more invasive surgical treatment.

The initial evaluation and management of patients with recurrent epistaxis should include the following:

• Anterior rhinoscopy and nasal endoscopy performed atraumatically, with careful removal of any eschar or blood clots, to assess the number, size, and morphology of telangiectasias. According to Mahoney and Shapshay [7], there are three definite vessel types associated with nasal telangiectasias in HHT: small, punctate isolated telangiectasias (type I), diffuse lesions associated with "feeder" vessels (type II), and large isolated AVMs (type III). Type I lesions are more likely to be associated with mild epistaxis, while type II and III lesions are often associated with moderate to severe epistaxis. Morphological typification and quantification of the number of lesions may be useful for treatment planning. For example, smaller type I lesions tend to respond well to KTP laser treatment [8], while larger type II and III foci are better treated with electrocauterization or coblation [7].

Epistaxis severity score: validated scoring tool used to quantify epistaxis severity. It includes queries related to epistaxis duration, frequency, intensity, attendance of anemia, the necessity for blood transfusion, and medical treatment [9].

• According to the Second International Guidelines for the Diagnosis and Management of HHT, it is recommended that all patients with HHT be screened for pulmonary and cerebrovascular AVMs at the time of diagnosis [2]. Bubble echocardiography is a screening test recommended every 5-10 years after initial screening to exclude the formation of new AVMs. If positive, bubble echocardiography should be followed by a chest CT scan to get a complete picture of the size and number of AVMs. Patients with pulmonary AVMs should be referred to a pulmonologist, interventional radiologist, or thoracic surgeon for treatment. Brain MRI is recommended at diagnosis, and referral to neurosurgery is necessary if cerebrovascular malformations are present. Routine gastrointestinal endoscopy is not indicated, but patients with a history of anemia disproportionate to the severity of gastrointestinal and/or epistaxis should undergo gastrointestinal endoscopy for the diagnosis and treatment of gastrointestinal AVMs.

• Treatment of ENT manifestations of Rendu-Osler-Weber syndrome is supportive, includes control and management of bleeding and is the same as management of non-HHT bleeding [1,3].

• Iron absence in combination with elevated transferrin, low hemoglobin, and ferritin is characteristic in patients with middling to severe epistaxis and may be due to gastrointestinal bleeding. It should therefore be tested regularly from the age of 35.

• Non-pharmacological preventive measures: Proper nasal hygiene and avoidance of triggers and anticoagulants if possible. Humidifying medicines can protect the integrity of the nasal mucosa. They prevent nasal crusting, reduce damage to the nasal mucosa, and reduce nosebleeds [1]. Nasal ointments usually contain lanolin, dexpanthenol, or saline [10].

• Nasal packing with hemostatic agents (Merocel, Surgicel, gelatin sponges) is the only conservative strategy for acute management of bleeding. Nasal packing is a safe option in the presence of bilateral bleeding; unlike coagulation, there is no risk of septal perforation. The main disadvantage is that its removal can cause re-bleeding [10].

• Pharmacological therapies include the following:

(1) Antifibrinolytics: reinforce coagulation and increase endoglin and ALK1 levels by inhibiting fibrinolysis associated with telangiectasia. Tranexamic acid is a strong anti-fibrinolytic agent that, according to the multinational ATERO study, significantly reduces the duration of epistaxis [11]. However, it is contraindicated in patients prone to thrombosis.

(2) Hormonal therapy: specific estrogen receptor modulators (SERMs). Estrogens, and in particular raloxifene and bazedoxifene, are transcriptional activators of ACVRL1/ALK1 and ENG gene promoters that cause a significant reduction in epistaxis frequency and quantity, as well as an improvement in the hemoglobin levels [12,13].

(3) Immunosuppressant: tacrolimus (FK506) is a medicine given to prevent rejection in a liver transplant after hepatic failure due to liver AVMs. It is also very effective in increasing the expression of ENG and ALK1, significantly reducing epistaxis, anemia, and telangiectasias [14]. Nonetheless, sequential use of FK506 is not recommended due to its suppressive effect on the immune system.

(4) Atorvastatin: in addition to its lipid-lowering effects, it also increases the expression of endoglin and the eNOS protein, thus improving epistasis [15]. However, secondary effects of statins (muscle pain, increased risk of diabetes, abnormal liver enzyme tests) should be considered.

(5) Antiangiogenic drugs: The current strategy to reduce bleeding in HHT is to reduce the excessive abnormal vasculature present in the nasal mucosa through anti-angiogenesis. VEGF protein levels appear to be elevated in patients with HHT. VEGF neutralization with bevacizumab has been shown to be beneficial in the treatment of patients with HHT associated with severe hepatic AVMs [16] or severe gastrointestinal hemorrhage [17]. Nevertheless, in two independent studies, used treatment with bevacizumab nasal spray did not decrease epistaxis duration in patients with HHT [18]. Thalidomide suppresses levels of angiogenic and growth factors (VEGF, TNF-α, and bFGF) and reduces epistaxis number and severity by stimulating vessel maturation through pericyte recruitment to vessel walls [19]. The b-blocker propranolol shows angiogenesis-inhibiting and apoptosis-promoting effects on endothelial cells, suggesting its topical use in HHT [20]. The use of antiangiogenic therapies, including bevacizumab and pazopanib to treat bleeding in HHT and rapamycin (sirolimus), PIK3CA (alpelisib), and MEK (trametinib) inhibitors in the treatment of complex vascular malformations is the focus of a very recent study by Al-Samkari et al. published in May 2022 [21].

(6) Antioxidants NAC: neutralize the harmful effects of reactive oxygen species (ROS) and thus may prevent or treat diseases related to oxidative stress. The use of NAC causes a significant reduction in the frequency and severity of epistaxis, notably in male patients and HHT1 patients with ENG mutation. In HHT2 and women patients with an ALK1 mutation, only a tendency toward melioration is remarked [22].

• Topical drug therapy: Several randomized clinical trials have taken place to evaluate topical instillation treatments that are associated with HHT given that 80%-90% of nasal telangiectasias are located in anatomic areas accessible for topical therapy and the desire to avoid the risks of systemic therapies. In a study conducted in North America by Whitehead et al., three pharmaceutical agents (bevacizumab, tranexamic acid 10%, estriol 0.1%) with different mechanisms of action were selected, and their effectiveness in reducing epistaxis was evaluated by assessing factors such as frequency, duration, and severity of epistaxis; hemoglobin and ferritin level; the need for transfusion; emergency department visit; and treatment failure [23]. All groups had a significant improvement in epistaxis severity score. However, there were no statistically significant differences between them regarding the above factors.

• Surgical and invasive treatment is available when conservative approaches and medical therapy fail. The following techniques are applied:

(1) Monopolar and silver nitrate cautery increase the likelihood of septal perforation and the tendency to worsen the severity of epistaxis and therefore they are not recommended [24].

(2) Bipolar electrocautery and electrosurgical plasma coagulation (coblation) are effective surgical treatments for HHT-related epistaxis. Bipolar electrocautery, alone or in addition to KTP laser coagulation, has an immediate effect in controlling epistaxis. It is preferred over monopolar electrocautery due to its improved accuracy and shallower depth of thermal injury [25]. According to a recent study by Dür et al. in 2021 [10], bipolar cautery is indicated for the management of acute unilateral epistaxis because it minimizes the risk of septal perforation. However, a faster return to the hospital with nasal bleeds was associated with the use of bipolar cautery. Coblation combines perfusion bipolar and coblation functions and is useful for the excision of large, clustered telangiectasias. It is also particularly useful in cases of active bleeding. Because it uses lower temperatures, there is less risk of thermal tissue damage; Joshi et al. found coblation to be a safe, effective, and well-tolerated treatment [26], and Luk et al. found it to be an acceptable alternative to KTP laser [8].

(3) Laser photocoagulation [27] (ND: YAG, KTP, Argon, CO2) can destroy defective vessels while minimizing the depth of thermal damage to the surrounding normal mucosa. It causes scarring and fibrosis, leading to a decrease in capillary dilatation. Laser therapy is effective in reducing the duration, frequency, and severity of epistaxis in HHT patients. However, the result is often time-limited, and the procedure must be repeated. Problems with the use of lasers are that not all flexible fiber devices can be used, and their maneuverability in the nasal cavity can be limited. Other disadvantages of laser treatment are their unavailability in some facilities and high cost. In addition, eye protection is essential for all ENT staff, and special precautions such as wet eye pads and towel drapes are required during use [28].

Abiri et al. reviewed 55 studies using different types of lasers to control epistaxis in patients with HHT and concluded that the ND: YAG laser gave better results, especially for severe epistaxis as compared to argon lasers [29]. However, the ND: YAG laser does have its disadvantages: it cannot be used to control acute bleeding and its bilateral application carries the risk of septal perforation.

In 2021, the Department of Otorhinolaryngology of the Policlinic San Matteo in Pavia, the reference center for the diagnosis and treatment of HHT in Italy, described its 20-year experience in the endoscopic surgical treatment of epistaxis in 323 HHT patients, including those with a history of severe epistaxis and blood transfusions. He concluded that endoscopic minimally invasive nasal surgical techniques, particularly argon plasma coagulation (APC), can be used as a first step [30]. These advantages include low morbidity, less trauma to the nasal mucosa, low risk of nasal septal perforation, high reproducibility of treatment, no need for postoperative nasal packing, the possibility of local anesthesia, and short hospital stay [30]. APC is an endoscopic cauterization method performed through a nozzle, that is, without direct contact with the lesion, using high-frequency electric current through ionized Arg gas [31]. The limited tissue coagulation depth (1-2 mm), the lower risk of perforation, the excellent results in tissue coagulation, and its easy execution make APC an alternative and safer method than ND: YAG laser.

(4) Endovascular embolization: In a study by Trojanowski et al., the success rate was 85%, but the recurrence rate was also high at 43% [32]. This treatment is mainly indicated in acute cases that do not respond to nasal packing. However, embolization is not recommended by most authors due to the high risk of stroke and blindness, especially when treating the anterior and posterior ethmoidal arteries simultaneously. Pulmonary AVMs, with feeding vessels larger than 2-3 mm in diameter, require catheter-based occlusion. The devices used for embolization are balloons, coils, and recently Amplatzer®. In special cases of cerebral AVMs where embolization is possible, occlusion can be achieved with onyx glue [1,3].

(5) Sclerotherapy is considered by Boyer et al., more effective than many other standard forms of treatment [33]. Injection of sodium tetradecyl sulfate or polidocanol [34] into the defective vessels results in scarring and fibrosis, decreased capillary dilatation, and ultimately reduced epistaxis. Significant improvements in both mean epistaxis severity score and quality of life have been observed. Possible side effects of sclerotherapy include tissue necrosis, injection site cellulitis, rarely anaphylaxis, pulmonary embolism, and permanent blindness due to occlusion of central retinal or ocular arteries [35]. The main advantage of sclerotherapy is the avoidance of general anesthesia, which is an important advantage for patients suffering from pulmonary and cardiac sequelae of HHT. Disadvantages include reduced access to posterior lesions and reduced ability to control massive bleeding in a wakeful patient.

(6) Cryotherapy is a procedure that involves applying subfreezing temperatures to various tissues and has been widely used in otolaryngology. The contact of the nasal mucosa with a temperature of -70°C leads to the denaturation of cellular proteins and the intracellular shift of electrolytes. As the endothelial cells react in a similar way, microthrombosis forms in the vessels and causes perfusion disturbance with subsequent sequential ischemia, temporary hemostasis with subsequent re-epithelialization of the nasal mucosa, and a decrease in the frequency of chronic recurrent bleeding over time [36].

(7) Nasal septal dermoplasty is recommended only as a last line of defense. In this case, the nasal mucosa is replaced with skin graft tissue or oral mucosa, but the underlying perichondrium is preserved. Harvey et al. showed that septal dermoplasty can reduce the need for multiple laser procedures by up to 57% [37]. However, telangiectasias have been found to grow through the skin graft, leading to recurrent epistaxis. While this technique allows adequate treatment of septal lesions, it does not control epistaxis when bleeding is from the lateral wall. The major complications are worsening of sinus infections, crusting, and decreased sense of smell [38]. Therefore, this treatment is recommended only in severe cases. In a study by Lesnik et al., this method was used in patients who developed septal perforation after other treatments [39].

(8) Nasal closure of one or both nasal cavities, known as the Young procedure, is used in patients who are transfusion-dependent or have severe life-menacing epistaxis or as a last resort when other treatment options have failed. It results in complete cessation of epistaxis ranging from 57-94% [40]. The aim of nasal closure is to prevent the airflow through the nose and the drying effect of the nasal mucosa as an irritant of telangiectasia. The operation is generally performed on both nasal chambers, especially when there is a septal perforation; otherwise, it may be confined to the worse side. However, it has significant side effects such as chronic mouth breathing, xerostomia, anosmia, and nasal voice, which greatly affect the quality of life. The authors' experience is that severe epistaxis can also occur after Young surgery, creating significant problems in dealing with acute bleeding when the nasal cavities are obstructed. For this reason, we no longer recommend the use of this specific operation.

Since January 2018, the Department of Otorhinolaryngology of Regensburg University has been using a care plan for HHT patients and their treating physicians, called the Osler Calendar [41]. Its aim is to inform patients about the most common symptoms of HHT, to record their frequency, and how to approach them. The Osler Calendar outlines the screening tests required, records the results, and reminds the patient of the necessary follow-ups. All treatment measures since the patient's last contact are recorded, including iron supplementation, blood transfusions, nasal packing, coagulation, and laser, surgical, and medical therapies.

Most patients with HHT who have adequate access to healthcare will have a normal life expectancy. There is a bimodal distribution of mortality, with peaks at age 50 and then from 60 to 79 [42]. Most of the mortality of HHT results from complications of AVMs, particularly in the brain, lungs, and GI tract.

This systematic review adhered to the PRISMA guidelines, ensuring a methodical approach. Relevant articles were identified through a comprehensive search of the PubMed bibliographical database employing specific keywords (i.e. hereditary hemorrhagic telangiectasia, Rendu-Osler-Weber syndrome, telangiectasias, recurrent epistaxis) to yield accurate results.

## Conclusions

HHT is a composite illness with a multitude of clinical manifestations that require management and treatment by a multidisciplinary team, including otolaryngologists, gastroenterologists, hematologists, pulmonologists, cardiologists, interventional radiologists, neurosurgeons, and genetic counselors. Despite the rarity of Rendu-Osler-Weber syndrome, otolaryngologists must be aware of the general and ENT manifestations of this syndrome so that they can suspect disease from the initial stages, document the diagnosis with the help of other specialties, and treat with particular attention to recurrent epistaxis, which are the most common and dominant manifestations of HHT.
